# Death Came While He Was Trying to Save

**Published:** 1899-05

**Authors:** 


### DEATH CAME WHILE HE WAS TRYING TO SAVE.

### Dr. Reuben Ludlam Stricken at the Critical Point of an Operation—His Son Finishes.

Chicago, April 29th.—Dr. Reuben Ludlam, Sr., President of Hahnemann Medical College and one of the most widely known homœopathic practitioners in the world, was stricken with heart disease to-day just at the critical point in an operation he was performing at the college hospital. Although Dr. Ludlam was carried from the operating room in a dying condition, his son, Dr. Reuben Ludlam, Jr., who had been acting as assistant, immediately seized the instrument from his father's hand and, to save the life of the patient, continued the surgical work.

Dr. Reuben Ludlam, Sr., expired within five minutes in a room adjoining the operating room, but it was not till a half hour later that the son, the patient having come safely through the operation, went to his side. The patient was a woman. The operation was the removal of a fibroid tumor of large size from the abdominal cavity. The patient probably will recover as the result of young Dr. Ludlam's service. —*The Times*, April 30th, 1899.

---

Drug Intemperance.—There is a source of nervous ailments entirely special to this age, and the unexpected outcome of our nineteenth century chemistry and advertising. Intemperance in drugs is becoming more common, and it may possibly outstrip the abuse of alcohol in its evil results. The manufacture of new chemical products is supplying the public with endless carbon derivatives of high molecular power, and of imperfectly known physiological action. Some are most dangerous, and their continued indulgence leads to confirmed neuroses or hopeless nerasthenia, and it thus comes to pass that as the therapeutic activity of the profession tends to abolish disease, that of the public is manufacturing it.

